# Priapism in Sickle Cell Anemia: Emerging Mechanistic Understanding and Better Preventative Strategies

**DOI:** 10.1155/2011/297364

**Published:** 2010-11-30

**Authors:** Genevieve M. Crane, Nelson E. Bennett

**Affiliations:** Institute of Urology, Lahey Clinic, 41 Mall Road, Burlington, MA 01805, USA

## Abstract

Sickle cell anemia is a common and disabling disorder profoundly affecting mortality as well as quality of life. Up to 35% of men with sickle cell disease are affected by painful, prolonged erections termed ischemic priapism. A priapic episode may result in fibrosis and permanent erectile dysfunction. The severity of sickle cell disease manifestations is variable dependent on a number of contributing genetic factors; however, priapism tends to cluster with other severe vascular complications including pulmonary hypertension, leg ulceration, and overall risk of death. The mechanisms underlying priapism in sickle cell disease have begun to be elucidated including hemolysis-mediated dysregulation of the nitric oxide signaling pathway and dysregulation of adenosine-mediated vasodilation. A better understanding of these mechanisms is leading toward novel preventative strategies. This paper will focus on the mechanisms underlying development of ischemic priapism in sickle cell disease, current acute and preventative treatment strategies, and future directions for improved management of this disorder.

## 1. Introduction

Sickle cell disease (SCD)  is a common disorder with 8% of African Americans heterozygous for the hemoglobin S point mutation (HbS) in the *β*-globin gene and approximately 50,000 Americans homozygous for this mutation [1, 2]. Hemoglobin is a tetrameric protein composed of two *α*-chains and two *β*-globin chains. Deoxygenation results in aggregation and polymerization of HbS tetramers, formation of needle-like hemoglobin fibers, and distortion of red cells into the characteristic sickle shape. 

 Sickling is dependent on multiple variables including hemoglobin concentration and hydration status, pH and degree of deoxygenation. Sickling is more common in vascular beds with low-flow states including the splenic sinusoids, the bone marrow, the penile corpora during an erection, or any inflamed tissue. The combined influence of these factors can result in a vicious cycle of inflammation, hypoxia, and acidosis resulting in increased sickling, vessel occlusion, and ischemia [3]. 

 Manifestations of SCD are widespread and generally secondary to ischemia from vessel occlusion and hemolysis from direct rupture of sickle cells that have lost normal red cell deformability. The type and severity of complications vary significantly between individuals. Common manifestations include vaso-occlusive crises, the result of hypoxic injury or infarction, which may affect the brain, pulmonary vessels, spleen, bone marrow, kidney, retina, penis or other tissues resulting in sequelae such acute chest syndrome with pulmonary infarction, stroke or splenic infarction. Splenic infarction and subsequent fibrosis ultimately contribute to increased susceptibility to infections from encapsulated bacteria leading to increased risk of septicemia and meningitis, the most common causes of death in children with SCD. Leg ulcers are another common manifestation of vaso-occlusion. A comprehensive discussion of the manifestations of SCD is beyond the scope of this paper. 

 This paper will focus on one common manifestation that can significantly affect quality of life of men with SCD, which is the development of prolonged, painful erections known as low-flow or ischemic priapism that result in tissue ischemia and attenuated or absent return of functional erections. Reports of the lifetime prevalence of ischemic priapism in SCD range from 2–35% [4, 5]. High flow priapism, which results from increased arterial flow generally caused by trauma, does not result in tissue ischemia and has not been associated with increased risk in SCD patients. The mechanisms underlying high and low-flow priapism completely differ, as do their treatments; therefore, this paper will focus only on ischemic priapism, which is substantially increased in SCD patients.

## 2. Pathophysiology of Priapism

In the flaccid state, the erectile tissue smooth muscle is tonically contracted as are the arterial and arteriolar vessels, allowing only a small amount of flow to meet nutritional needs. When neurotransmitters are released to signal an erection, there is smooth muscle relaxation resulting in filling of the sinusoids and ultimate compression of venous outflow resulting in trapping of blood. This results in an erection with pressure greater than that of systolic blood pressure. The corpora cavernosa are covered in a tunica albuginea, a bilayered structure with an inner layer that supports the cavernous tissue and an outer layer that runs longitudinally from the glans to insert onto the pubic rami. Emissary veins run between the inner and outer layers. During an erection, these are compressed along with the subtunical venous plexus to prevent venous outflow. The corpora spongiosum lacks the tunica, and therefore, serves as an arteriovenous fistula with less rigidity than the corpora cavernosa [6]. The principal neurotransmitter for erection is nitric oxide (NO), which is released by both nerve terminals and endothelial cells. The NO then diffuses into both trabecular cells and arterial smooth muscle cells. This activates guanylate cyclase, which catalyzes the formation of cGMP from GTP, and results in a cascade that decreases intracellular calcium and opens potassium channels to cause smooth muscle relaxation. Alternative pathways generate cAMP, which enhances this effect. Detumescence follows degradation of cGMP and cAMP. cGMP is degraded by phosphodiesterase-5 (PDE5), the target of sildenafil, vardenafil and tadalafil. 

 Stasis and low blood flow rates within the sinusoids of the erectile tissue make the penis a site at high risk for developing a veno-occlusive crisis in SCD. Ischemic priapism is characterized by a prolonged erection that is usually painful and not associated with sexual excitement or desire. Typically, the corpora cavernosa are tense, congested and tender to palpation while the glans and corpus spongiosum are usually soft and uninvolved. Classically, the primary mechanism is thought to be obstruction of venous drainage resulting in viscous, hypoxic blood [7] leading to interstitial edema and fibrosis of the cavernosa, ultimately resulting in impotence. The length of time of ischemia directly correlates with the likelihood of return of erectile function, and therefore, ischemic priapism is considered a urologic emergency. For example, in one study, all patients with ischemic priapism episodes of greater than 12 hours duration had reduction in erectile rigidity. No patients with priapism greater than 36 hours had return of spontaneous functional erections [4].

## 3. Mechanisms Underlying Sickle Cell-Induced Priapism

While anatomic factors and a low-flow state contribute to making erectile tissue prone to sickle cell-induced pain and ischemia, abnormal regulation of the nitric oxide (NO) pathway and its downstream signaling may be particularly key in the development of priapism and other vascular complications in SCD. 

**Figure 1:**
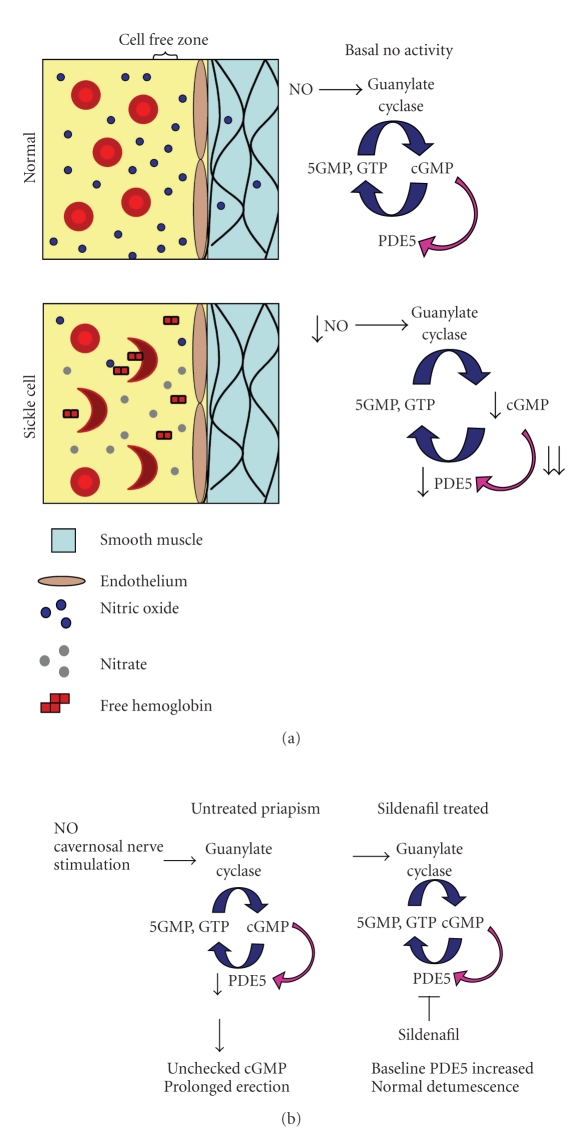
Abnormal nitric oxide signaling leads to priapism in SCD. NO can be readily scavenged by free hemoglobin and deactivated by conversion to nitrate. This is normally prevented by compartmentalization of hemoglobin in red cells and a cell free zone adjacent to the endothelium ((a), first panel adapted from Liao [9] and Rother et al. [23]). In SCD, chronic hemolysis leads to higher levels of free hemoglobin and decreased NO availability. This results in decreased basal levels of its downstream effector, cGMP, and decreased PDE5, which degrades cGMP. Following cavernosal nerve stimulation in SCD (b), normal levels of cGMP are achieved as NO is released from nerve terminals. However, PDE5 levels remain low, leading to prolonged cGMP activity and prolonged erection. This can be prevented by long-term treatment with sildenafil ((b), second panel). Inhibition of PDE5 by sildenafil results in increased cGMP, which then increases basal PDE5 levels through normal feedback mechanisms [13, 14].

 NO is a potent vasodilator and key to tumescence as described above, but free NO is readily scavenged in the blood by hemoglobin. It is converted to nitrate plus methemoglobin following reaction with oxyhemoglobin and to iron-nitrosylhemoglobin following reaction with deoxyhemoglobin [8]. The ability of hemoglobin to scavenge NO is normally dramatically reduced by its compartmentalization within red blood cells, which creates a cell-free and therefore hemoglobin-free zone adjacent to the vessel endothelium due to laminar flow (Figure 1(a)). In addition, the presence of the cell membrane, cytoskeleton and other factors increase the diffusion distance between NO and hemoglobin. As a result, hemoglobin contained in red cells has less than 0.2% of the scavenging capacity of free hemoglobin [9]. The chronic intravascular hemolysis in SCD changes this tight regulation of NO. SCD patients have elevated free hemoglobin concentrations compared to normal volunteers [8], and these concentrations were frequently sufficient to blunt vasodilation in response to nitroprusside administration. Pain crises may be exacerbated by further hemolysis, NO scavenging and prevention of vasodilation in the presence of ischemia. 

 Other studies have also associated risk for priapism with markers of hemolysis. Lactate dehydrogenase (LDH), bilirubin and reticulocyte count were all elevated in men with SCD who experienced priapism compared with men with SCD who had never experienced an episode of priapism [10]. In another study, LDH level closely correlated with the free-hemoglobin level in plasma, accelerated NO consumption and impaired vasodilation [11]. In addition, LDH levels in this study could be used to identify a subset of SCD patients at higher risk for vascular complications including pulmonary hypertension, priapism, and leg ulcers. 

 Chronic scavenging of NO results in a decreased expression of downstream regulatory molecules including PDE5 that normally degrades cGMP, the second messenger in NO signaling. Genetically modified mice homozygous for the sickle cell mutation (SS^−/−^) have been shown to express decreased levels of PDE5 in penile tissue, and similar effects were seen in mice deficient for endothelial nitric oxide synthase (eNOS^−/−^), which also develop priapism [12]. Nonetheless, following cavernosal nerve stimulation, these mice express high levels of cavernosal cGMP, likely secondary to the fact that NO released from nerve terminals is not as readily scavenged. The combination of chronically decreased PDE5 and normal cGMP generation following nerve stimulation may result in an unregulated, prolonged erection. 

There is complex regulation of PDE5 by cGMP and its effector, cGMP-dependent protein kinase (PKG) [13]. cGMP is the substrate for PDE5, but it can also bind at an additional allosteric site to alter the conformation of PDE5, exposing a phosphorylation site. This site can be phosphorylated by PKG, which then increases the activity of PDE5. Also, as a normal feedback mechanism, the promoters which regulate the expression PDE5 isoforms found in penile tissue are responsive to increasing concentrations of cGMP [14]. That is, cGMP stimulates PDE5 expression to regulate its own degradation, suggesting that PDE expression could be enhanced by agents that increase cGMP levels. This phenomenon has now been demonstrated both in vitro and in vivo. Culture of cavernous smooth muscle cells treated with sildenafil increased PDE5 expression, and young but not old rats treated with sildenafil for 3 weeks demonstrated an elevation of PDE5 expression in penile tissue following treatment [15].

The ability of elevated cGMP to stimulate increased expression of PDE5 has raised the possibility of a novel preventative strategy for priapism in SCD. While initially somewhat paradoxical, these results suggest that treatment of sickle cell patients with PDE5 inhibitors may actually help prevent priapism by correcting the chronically low levels of PDE5 in their cavernosal tissue (Figure 1(b)). Indeed, Burnett and colleagues who posed this hypothesis have found that long-term, low-dose PDE5 inhibitor treatment may help reduce priapism in a series of cases [13]. 

Additional pathways may also affect the development of priapism in SCD. One that has recently gained interest is the regulation of adenosine signaling. Similar to NO, adenosine is a potent vasodilator and neurotransmitter. Adenosine signals through G-protein coupled receptors to increase cAMP to result in smooth muscle relaxation, and intracavernosal injection of adenosine can be used to induce erection. Studies in adenosine deaminase deficient mice, which develop prolonged erections, have demonstrated that excessive adenosine accumulation and adenosine receptor signaling contribute to priapism through smooth muscle relaxation [16]. Adenosine signaling was similarly elevated in the sickle cell SS^−/−^ mouse model. Elevated levels of adenosine appear to contribute to fibrosis, as adenosine deaminase enzyme therapy attenuated fibrosis in both mouse models [17]. These results suggest that the adenosine signaling pathway may be an excellent target for the development of new treatment strategies to prevent priapism or to limit fibrosis following a priapic episode. 

In addition to pathways that regulate vasodilation, appropriate regulation of vasoconstriction is also necessary for normal erectile function. RhoA and its downstream effectors, Rho-kinases (ROCK1 and 2), help maintain the penis in a flaccid state and have been shown to have abnormal activation in erectile dysfunction [18] including in old age and diabetes. There is reduced activity of components of this pathway in both eNOS^−/−^ and SS^−/−^ mice with priapism [19, 20]. Additional work will be required to further clarify the role of this pathway in priapism as well as appropriate targets for therapy. 

## 4. Treatment Strategies

Acute treatment for ischemic priapism should be instituted within hours given the increasing likelihood of cavernosal fibrosis and permanent erectile dysfunction. Standard treatment involves penile blood gas studies, corporeal aspiration, and phenylephrine injection to induce smooth muscle contraction and detumescence. SCD patients may benefit from early high-dose intracavernosal phenylephrine given that the acidic pH of the ischemic cavernosa may decrease the affinity of adrenergic receptors for their ligands [21]. If conservative measures fail, penile shunt surgery should be performed. This may involve anastamosing corpus spongiosum and corpora cavernosa to create an internal fistula. SCD patients may also benefit from hydration, blood transfusions, exchange transfusions, or hyperbaric oxygen. 

Ideally, patients at high risk for the development of such episodes would be identified early to increase education of the risks of prolonged priapism and to institute potential prophylactic strategies. Increased awareness of the signs of priapism and education on the need for early treatment may be the best way to prevent longterm sequelae. Despite the risks of ischemic priapism and its association with SCD being well known in the medical community, few men receive education on the need to seek urgent medical attention for this issue [4]. In addition, it may be possible to identify the subset of SCD patients at highest risk for severe priapic episodes for education and prophylatic strategies before permanent damage has taken place. This is because prolonged episodes of priapism that result in intracavernosal fibrosis are often preceded by periods of “stuttering priapism” (72% of cases in one report [5]) or acute transient attacks as originally described by Hinman [7]. Treatment of individuals with stuttering priapism with PDE5 inhibitors has been reported to reduce the frequency of these episodes [13]. Use of PDE5 inhibitors to abort acute attacks has also been reported [22], but positive results would be less likely to be attributable to short-term changes in PDE5 levels and, in fact, such use could potentially worsen a priapic event [13]. However, it has been hypothesized that use of PDE5 inhibitors in acute attacks may result in vasodilatation of cavernosal tissue allowing egress of sickle cells. 

There has been evidence to suggest that nocturnal oxygen desaturation, particularly in children, may contribute to the onset of painful crises [24, 25]. This could be particularly relevant to priapism given the normal physiology of nocturnal tumescence, and patients may benefit from prophylatic treatment including nocturnal oxygen or the use of continuous positive airway pressure machines. 

Hormonal therapy has also been used to decrease the incidence of priapism [26] including GnRH analogs, stilbestrol [27] and antiandrogens which are associated with significant side effects including hot flashes and gynecomastia. More recently, one group has investigated the use of finasteride, a 5 *α*-reductase inhibitor, to decrease the frequency of priapism in a population with SCD and recurrent priapism with a significant decrease in the number of episodes per month [28]. Unfortunately, these hormonal therapies may have significant or unknown effects on fertility and libido, posing a significant disadvantage for use in the typically young SCD patient population.

Long-term use of oral [29, 30] or intracavernous delivery [31] of *α*-adrenergic agonists has also been investigated with some success as first described by Virag and colleagues. Digoxin has been used and is believed to exert its effect by inhibition of the Na/K ATPase pump to prevent relaxation of cavernosal smooth muscle. Neuromodulatory agents have also been used, particularly in spinal cord patients, to moderate the autonomic and somatic reflex pathways involved in erection. These include gabapentin, a synthetic analogue of GABA, an inhibitory neurotransmitter, and baclofen, a GABA receptor agonist. A detailed paper of these and other agents in the chronic treatment of ischemic priapism can be found in a recent paper by Chow and Payne [26]. 

If irreversible damage has been done, long-term management of erectile dysfunction may be treated with placement of a penile prosthesis to restore function depending on the preference of the patient, likelihood of success and a detailed discussion of the risks of the procedure. A severe and prolonged episode of priapism will result in cavernous smooth muscle necrosis, fibrosis, and ultimately penile shortening. If erectile function is unlikely to recover, immediate implantation of a penile prosthesis may, therefore, be considered to avoid the complications of penile fibrosis and shortening [32]. 

 The type and severity of the complications of SCD are variable depending on a variety of modulating genetic factors including the presence of other types of hemoglobin, particularly persistent fetal hemoglobin (HbF). Normally after birth, a switch occurs in the *β*-globin locus from production of predominantly *γ*-globin to *β*-globin. Hydroxyurea, which blocks DNA synthesis, has been used to help induce HbF in the adult, thus helping to reduce the frequency of sickle cell crises; however, it results in significant side effects including anemia, neutropenia, and renal and liver dysfunction.

## 5. Conclusions

While the complications associated with the painful crises associated with sickle cell disease including ischemic priapism remain common and debilitating in the SCD population, a better understanding of the underlying biology is emerging. There is hope for new preventative treatments including long-term low-dose PDE5 inhibitors to normalize NO downstream signaling and potential inhibition of adenosine signaling to minimize fibrosis following an episode of priapism.
